# The Relationships between a Dietary Pattern Linked to Cardiometabolic Risk Factors and Life Satisfaction in Early Adolescence

**DOI:** 10.3390/ijerph17155489

**Published:** 2020-07-29

**Authors:** Geeta Appannah, Nor Aishah Emi, Wan Ying Gan, Zalilah Mohd Shariff, Nurainul Hana Shamsuddin, Azriyanti Anuar Zaini, Mahenderan Appukutty

**Affiliations:** 1 Department of Nutrition and Dietetics, Faculty of Medicine and Health Sciences, Universiti Putra Malaysia, Serdang 43400, Selangor, Malaysia; aishahemi292@gmail.com (N.A.E.); wanying@upm.edu.my (W.Y.G.); zalilahms@upm.edu.my (Z.M.S.); 2 Department of Family Medicine, Faculty of Medicine and Health Sciences, Universiti Putra Malaysia, Serdang 43400, Selangor, Malaysia; nurainul@upm.edu.my; 3 Department of Paediatrics, Faculty of Medicine, University of Malaya, Kuala Lumpur 50603, Malaysia; azriyanti@ummc.edu.my; 4 Sports Science Programme, Faculty of Sports Science and Recreation, Universiti Teknologi MARA, Shah Alam 40450, Selangor, Malaysia; mahen@uitm.edu.my

**Keywords:** dietary patterns, life satisfaction, adolescents, Malaysia

## Abstract

Little is known about the contribution of dietary patterns of poor quality on life satisfaction among Malaysian children. We evaluated associations between an empirically derived ”high sugar, high fibre, high dietary energy dense (DED) and low fat” dietary pattern and life satisfaction score in adolescents. A total of 548 adolescents aged 13 years were recruited from randomly selected public schools located in three southern states of Peninsular Malaysia. Dietary intake was assessed using a validated food frequency questionnaire (FFQ) while life satisfaction was measured using a Multidimensional Students’ Life Satisfaction Scale (MSLSS). Z-score for a ”high sugar, high fibre, high DED and low fat” dietary pattern was estimated by applying reduced rank regression analysis. Relationships between the dietary pattern and life satisfaction scores were assessed using regression models. Mean and SD of life satisfaction score was higher in girls (70.5 (12.8)) compared to boys (67.6 (15.4)), *p* < 0.05. The overall life satisfaction score (*β* = −0.119; 95% CI: −0.125, −0.004) was inversely associated with dietary pattern z-score as well as scores for self (*β* = −0.13; 95% CI: −0.170, −0.015) and living environment (*β* = −0.12; 95% CI: −0.163, −0.007) domains in girls. An opposite trend was observed for school domain in boys whereby an increasing dietary pattern score was positively associated with increasing life satisfaction score (*β* = 0.216; 95% CI: 0.054, 0.36). The finding of this study highlights the role of free sugar and DED particularly, within the framework of whole diet, and target population at risk to improve life satisfaction among adolescents.

## 1. Introduction

Life satisfaction is an imperative factor for good health and is demarcated as the cognitive judgment of an individual’s overall quality of life or with its certain domains [1]. While not conclusive, evidence on the correlates and predictors of higher life satisfaction in adults included higher socioeconomic status, healthy life-partner relationship, social support, paid-job and healthy financial status, good physical health and certain personality traits [2,3,4]. On the other hand, psychological processes such as lack of positivity, emotional and stress management skills could reduce one’s overall life satisfaction and therefore, may influence the development of mental health illnesses over a period of time [5,6,7,8]. Although life satisfaction is adaptive and not a permanent trait of one person, many life circumstances often remain consistent over time and therefore, to a certain extent, the formation of definite life satisfaction cognitive judgments early in life may track into adulthood [9].

Adolescence is a period of significant development and is marked with rapid biological, physiological and psychological changes. As such, the promotion of mental health should be given close attention during this stage of life. It was suggested that the majority of adult mental health problems commenced during adolescence indicating vulnerability in this period for the progression of mental disorders [10]. In 2011, the National Health and Morbidity Survey (NHMS) in Malaysia indicated that 24% adolescents aged between 13 and 15 years have mental health problems such as developmental disability, emotional and behavioral disorders [11]. These NHMS 2011 findings were consistent with global epidemiological data whereby one in five children were suggested to develop some form of mental illnesses by early adulthood [12]. Nonetheless, some studies have indicated that there are quite a number of young people who do not meet the diagnostic criteria of mental illnesses (subthreshold) during adolescence were found to have mental health problems later in life [13,14]. Hence, identifying risk factors that may influence mental illnesses including life satisfaction in young people may suggest effective prevention strategies.

Parallel to the increasing prevalence of mental illnesses, the proportion of obesity and other cardiometabolic risk factors were also magnified among Malaysian young people over the last three decades [15,16]. It may be possible that lifestyle predictors of obesity and other cardiometabolic risk factors may also influence mental illnesses in adolescents. Unhealthy dietary patterns, for example, have been associated with mental illnesses in children and adolescents in the majority of Western countries [17]. Furthermore, a study conducted in Western Australian suggested adiposity and inflammation pathways as the plausible mechanisms for the prospective association found between a ”Western” dietary pattern characterized by high intake of takeaway foods, confectionary and red meat and mental illnesses in young people [18]. 

While adherence to a specific dietary pattern may play a role in the development of mental illnesses, there is a dearth of evidence on the relationship between dietary patterns and life satisfaction in young people. Previously, we found a positive relationship between a dietary pattern characterized by foods high in free sugar, dietary energy density (DED), fiber and low in fat and dyslipidemia as well as other cardiometabolic risk factors such as total cholesterol and low-density lipoprotein (LDL) cholesterol in adolescents aged 13 years in Malaysia [19]. Furthermore, we also found that 32% and 24% of the study adolescents to be either overweight or obese and dyslipidemic, respectively [19]. Since there is a possibility of tri-directional association between adiposity, inflammation and mental health, we aimed to assess cross-sectional relationships between poor life satisfaction as a potential indication for early markers of mental illnesses and a dietary pattern linked to cardiometabolic risk factors in the sample of adolescents aged 13 years in Malaysia. 

## 2. Materials and Methods 

### 2.1. Study Sample

The details of this study were previously described [19]. In brief, a total of 933 adolescents aged 13 years were enrolled from a total of 21 randomly selected public schools located in three southern states of Peninsular Malaysia in 2016. Adolescents from single ethnicity or gender type of schools, in other words, boarding and religious or vernacular were excluded from this study to ensure sex and ethnic representatives. The conduct of this study was fully approved by the Ethics Committee for Research Involving Human of Universiti Putra Malaysia [Reference No.: FPSK (EXP16) P031]. Additional approvals were obtained from the Ministry of Education Malaysia, respective state education departments as well as the selected schools. Furthermore, informed consent was obtained from the parents or primary caregivers before any recruitment was conducted. 

### 2.2. Dietary Assessment 

Dietary intakes in the past 12 months were assessed using a validated semi-quantitative MyUM adolescent food frequency questionnaire (FFQ) [20]. This FFQ was designed especially for adolescents aged 13 to 18 years and consisted of 195 food items. Detailed step-by-step instructions on how to fill in the FFQ were given to the study adolescents by a study dietitian. A flipchart and household measurement tools were used to aid the estimation of portion sizes. For each food item, the average frequency of consumption over the past year was recorded as “never”, “1–3 times per month”, “one time a week”, “2–4 times per week”, “5–6 times per week”, “one time a day”, “2–3 times per day”, “4–5 times per day”, or “≥6 times per day”. The completed FFQs were checked by a study researcher upon submission to ensure that all fields were filled in. The selected frequencies for each food item were converted to daily intakes and subsequently to daily nutrient intake using Nutritionist Pro software version 3.1 (First Databank Corporation, Axxya Systems, Stafford, TX, USA). This software comprised of the Malaysian Food Composition data and other international food items [21]. Adolescents who reported an overall energy intake outside the range of 400–8000 kcal were considered as outliers and hence, excluded from this study [22]. 

Since dietary misreporting is very common among adolescents, this was evaluated in this study. The estimation of dietary misreporting was done using a standardized equation [23]. This equation was based on energy intake (EI) to total energy expenditure ratio and its 95% confidence limit cut-offs were used to identify dietary misreporting [21]. The variable of dietary misreporting comprised of under, plausible and over-reporting and was used as a potential covariate in this study.

### 2.3. Dietary Patterns

The identification of a dietary pattern characterized by food intakes high in free sugar, fiber, DED and low in fat was previously discussed [19]. The identified dietary pattern was positively associated with a few indicators of cardiometabolic risk factors, particularly serum lipids [19]. In brief, the dietary pattern in this study was characterized using reduced rank regression analysis (RRR), a data-dimension reduction method that identifies dietary patterns that are specifically relevant to the health outcomes of interest by including a priori knowledge. A total of 195 food and beverage items in the FFQ were assigned to 13 predefined food groups and used to estimate response variables. These food groups (g/d) were included as predictor variables while intakes of DED, percentage of energy from total fat and free sugars, and fiber density were incorporated as response variables in the RRR model. Prior to this study, two pregnancy cohort studies in the United Kingdom and Australia reported significant associations between abovementioned response variables and cardiometabolic risk factors (including adiposity) during adolescence [24,25]. DED was estimated by dividing total food energy (kJ) by total food weight (g), without beverages [26]. Fiber density was determined by absolute fiber intake (g/d) divided by total daily energy intake (MJ). Percentage of energy from total fat intake and free sugar was expressed by dividing total energy intake from fat (kJ) and free sugar (kJ), respectively, by total energy intake (kJ) and then multiplied by 100. Dietary sugar was defined as all short-chained carbohydrates known as monosaccharide and disaccharides presented naturally in foods such as fruits or in manufactured products such as refined sugar [27]. The identified “high sugar, high fibre, high DED and low fat” dietary pattern in this study was depicted by high intakes of sugar-sweetened beverages (SSB), fruits, sweets as implied by their positive factor loadings and low intakes of cereal, cereal based dishes and meat and meat dishes as signified by their negative factor loadings [19]. Each respondent obtained a z-score for the identified dietary pattern and it reflected how their dietary intake paralleled to the identified pattern. 

### 2.4. Life Satisfaction

An abridged 18-item version of Multidimensional Students’ Life Satisfaction Scale (MSLSS) by Sawatzky et al. was used to measure adolescents’ life satisfaction, in this study [28]. The MSLSS was originally developed by Huebner in 1994 [29]. The items in the MSLSS corresponded to five specific life domains including family, school, friends, self and living environment. Adolescents were instructed to rate the 18 items based on their life experiences using a 6-point Likert scale, ranging from completely disagree to completely agree. Items in the same domain were summed up to represent scores for each domain whereas overall score was obtained by summing up all 18 items. A higher score suggested a higher life satisfaction. The MSLSS was previously used in a study conducted among adolescents aged between 14 and 17 years in Selangor, Malaysia, and showed moderate to strong reliability coefficients for each domain as well as in overall scale [30]. 

### 2.5. Potential Covariates 

Body weight was measured to an accuracy of 0.1 kg using a digital scale (Tanita HD319, Tokyo, Japan) while body height was measured to 0.1 cm using a SECA body meter (Model 213, Hamburg, Germany). BMI was calculated by dividing the measured body weight (kg) by the squared measured body height (m^2^). The 2007 WHO AnthroPlus Software (version 1.04) was used to estimate age and gender specific BMI z-scores. Overweight and obesity were defined as a BMI z-score more than one and two standard deviations above the WHO growth standard median, accordingly. The location of the selected schools was classified as either urban or rural based on the definition given by the Ministry of Education, Malaysia. Information on maternal education was obtained using a questionnaire sent to the parents or caregivers. The level of physical activity was assessed using a 10-item Physical Activity for Older Children (PAQ-C) questionnaire [31]. 

### 2.6. Statistical Analysis

Descriptive variables were presented in mean ± standard deviation [17] for continuous data and frequency (*n*) and percentage (%) for categorical data. Independent t-test and chi-square test were performed for comparison between genders. An association between life satisfaction and the identified dietary pattern was assessed using a regression model. Linear regression analysis was conducted by using the life satisfaction score as the dependent variable and dietary pattern z-score as the independent variable. The regression model was adjusted for potential covariates including school location (rural and urban) and maternal educational level in Model 1. In addition to that, adjustments in Model 2 comprised of those variables in Model 1, physical activity and BMI z-score. The final model consisted of those variables in Model 1 and 2 as well as dietary misreporting (Model 3). All analyses were run using IBM SPPS Statistics (IBM Corporation, New York, NY, US). A *p*-value of <0.05 was considered significant.

## 3. Results

### 3.1. Characteristics of the Study Adolescents

A greater number of girls compared to boys participated in this study (Table 1). The mean and SD of the overall life satisfaction score was 70.5 (12.8) in girls and 67.6 (15.4) in boys, *p* < 0.05. Similarly, means and SD for domains of life satisfaction score were generally higher in girls than boys and these were statistically significant for school and friends domains (*p* < 0.001). A higher “high sugar, high fibre, high DED, and low fat” dietary pattern score was observed in girls (0.06 (1.40)) compared to boys (−0.15 (1.14)). Boys were more physically active compared to girls as assessed using PAQ-C (2.8 vs. 2.4; *p* < 0.001). The majority of the adolescents’ mothers attained their education at secondary school level. This study was predominantly composed by adolescents recruited from schools located in rural areas.

### 3.2. Life Satisfaction

Table 2 describes the study adolescents’ responses to each item in the MSLSS. The 6-point Likert scale was categorized into two groups; “agree” and “disagree”. The selection of positive responses such as “strongly agree”, “moderately agree” and “mildly agree” was classified as “agree” while the selection of negative responses including “strongly disagree”, “moderately disagree” and “mildly disagree” was categorized as “disagree”. Almost all items in the questionnaire were agreed to (>80%) by the majority of the adolescents, except for two items in the domain of self. Specifically, item number 10 (“I think I am good looking”) was just agreed by 68.2%, while item number 12 (“Most people like me”) was agreed by 66.5%.

### 3.3. Relationships between a ”High Sugar, High fibre, High DED, and Low Fat” Dietary Pattern and Life Satisfaction 

Relationships between the identified dietary pattern and life satisfaction score are presented in Table 3. An inverse significant association was found in girls (β = −0.119; 95% CI: −0.125, −0.004) in Model 3, after adjusting for all the potential covariates. This indicates that for every 1 point SD increase in dietary pattern z-score, there was a significant 0.13 reduction in life satisfaction score in girls. Furthermore, inverse relationships were also observed between self and living environment domains and the identified dietary pattern, only in girls, in all the adjusted models. These associations became more apparent after adjusting for all potential covariates including dietary misreporting (Model 3). For every 1 point SD increase in the dietary pattern z-score, there was a significant 0.13 (95% CI: −0.170, −0.015) and 0.12 (95% CI: −0.163, −0.007) reduction in score for self and living environment, respectively, in girls, in Model 3. The opposite was observed in boys whereby an increasing dietary pattern score was positively associated with increasing life satisfaction score for the school domain in all the models. In the model where adjustment for all covariates was done (Model 3), an increasing score for school domain was observed for every SD increase in dietary pattern z-score (β = 0.216; 95% CI: 0.054, 0.364). 

## 4. Discussion

In this study, an increasing score for a dietary pattern characterized by food intakes high in free sugar, DED, fiber and low in fat was cross-sectionally associated with declining overall life satisfaction score as well as self and living environment domains, in girls. These associations were apparent mainly after adjusting for a wide range of covariates including school location, maternal education, dietary misreporting, physical activity and BMI z-score.

To date, no studies have assessed relationships between empirically derived adolescent dietary patterns and life satisfaction per se. Nonetheless, increasing rates of psychological illnesses were found to be inversely associated with poorer quality of diet in young people. Therefore, we have attempted to compare our findings to those studies on poorer diet quality and range of psychological illnesses indicators such as quality of life, unhappiness, emotional and behavioral problems in young people. Having said that, direct comparisons are limited due to the differences in the outcomes of the studies being compared and therefore, should be interpreted cautiously. For instance, a two-year prospective study among Australian adolescents suggested that a poorer diet quality assessed as lower adherence to the Dietary Guidelines for Children and Adolescents in Australia and a greater consumption of processed food in 2005 to 2006 was associated with reductions in the Pediatric Quality of Life Inventory (PedsQL) score in 2007 to 2008 [32]. On the other hand, an improvement in “Healthy” diet scores over the two-year period was associated with enhancement in PedsQL scores, in this study [32]. Findings from another adolescent study that has assessed mental health using PedsQL in New Zealand corroborate those found in Australian adolescents [33]. It was reported that healthy eating behaviors (no meal skipping and eating vegetables and fruits) were associated with better emotional health and the opposite was found between unhealthy eating behaviors and emotional distress, in this New Zealand study [33]. Similarly, a cross-sectional study of adolescents aged 14 years from a pregnancy cohort in Western Australia in 2009 found that a greater adherence to a ”Western” dietary pattern was significantly associated with poorer emotional and behavioral problems as represented by the Child Behaviour Checklist (CBCL) [34]. Additionally, a study in Canada reported that a higher diet quality assessed using Diet Quality Index-International (DQI-I) was inversely associated with children’s feelings of worrying, sadness and unhappiness [13].

There are several plausible mechanisms underlying the associations found in the current study. Although the identified dietary pattern was characterized using a priori information hypothesized to be associated with cardiometabolic risk factors, we found that the dietary pattern was not only explained by food intakes high in free sugar and DED but also foods high in fiber and low in fat. However, the contributions of foods high in fat were rather small, based on their correlation coefficient towards the identified dietary pattern (*r* < 0.2) [19]. Fibre, on the other hand, was contributed by high intakes of fruits, mainly of those tropical fruits known to be very sweet. A sensitivity analysis using three response variables hypothesized to be associated with obesity such as, DED, fiber density and percentage of energy from total fat also indicated a strong correlation between SSB and the overall dietary pattern (data not shown). In the current study, the contribution of free sugar mainly found in beverages and DED in sweets were most likely to explain the associations found between the dietary pattern and life satisfaction scores as they represent a typical “Western” diet. Some studies have suggested that ”Western” diets characterized by foods and beverages high in glycemic load, in other words, SSB, confectionery, sweets and candies may lead to greater levels of plasma C-reactive protein (CRP) [13,35]. CRP is a marker of general low-grade inflammation and high levels of this marker are common in adolescents, in parallel with the increasing prevalence of obesity and metabolic syndrome [32]. Inflammation, a process accompanied by increased oxidative stress has been suggested to facilitate psychological distress and depression in adults [36,37]. In this study, the direct relationships between the identified dietary pattern and life satisfaction components were more pronounced after adjusting for a range of covariates, including BMI z-scores. This indicates that modifying diets within the framework of lifestyle intervention may improve life satisfaction among adolescents, regardless of their body weight. Notwithstanding, future works are needed that consider overall dietary pattern and objectively measured current health status (e.g., cardiometabolic risk factors including obesity) to identify potential mechanisms and develop effective strategies or interventions.

The consumption of refined carbohydrates such as sweets and SSB may also have a direct effect on neurotropic factor [38]. This was suggested in an animal study by which a ”Western” style diet, especially one high in refined sugar lowered the brain-derived neurotropic factor (BDNF) levels rather rapidly in rats [39]. Similarly, a review of clinical studies suggested that BDNF is crucial for preventing poor mental health as it protects neurons from oxidative stress and promotes neurogenesis [40]. Evidence of higher intakes of sugar as well as fat and pathologies of various mental illnesses were previously reported in cross-sectional studies in adolescents [41,42]. Since life satisfaction and mental illnesses are linked to each other, it is plausible that inflammation and neurotropic factor may be associated with early markers of mental illnesses such that poor life satisfaction through the framework of high sugary and energy dense food intakes. 

Interestingly, boys who exhibited increasing scores for the school domain generally had higher scores for the identified dietary pattern. This co-occurrence implied the complexities of health-related behaviors as they do not necessarily distinguish in a different direction. It was presumed that people who adhere to a specific health-related behavior would intentionally compensate for the lack of another. In the context of this study, it may be that the high life satisfaction experienced at school could be due to exposure to unhealthy foods which are often more palatable compared to those foods consumed at home as well as due to connections and interactions with peers.

The main limitation of the present study is the cross-sectional nature of the data and that a causal relationship linking the identified dietary pattern and life satisfaction cannot be established. Therefore, the consistency of the findings observed in this study should be further evaluated in longitudinal analyses for establishing a causal effect. Another possible limitation of the study is that both FFQ and MSLSS were self-administered, thus reporting bias might have taken place. However, we have attempted to reduce the bias by giving examples on how to fill in the questionnaires and checking the completed questionnaires for any erroneous feedbacks. Furthermore, the regression models were also adjusted for dietary misreporting of variables to account for potential confounding factors due to bias in dietary assessment. Besides that, it is possible that the study sample was not representative of the wider Malaysian adolescent population, being drawn from three southern states of Peninsular Malaysia. Therefore, it may limit the generalizability of the study findings. Although we have attempted to control for a range of covariates in the analyses, we cannot however, rule out other uncounted variables.

Despite these limitations, to our knowledge this is the first population-based study to evaluate mental health assessed by life satisfaction scores and combined high-sugar and DED dietary pattern that was derived using RRR in an adolescent population in Malaysia. Other strengths of this study include the ability to adjust for a range of potential confounding variables including dietary misreporting, the use of valid and reliable measure of life satisfaction indicator for adolescents and the analyses of whole diet linked to cardiometabolic risk factors as compared to an analysis of individual food group or nutrient. 

## 5. Conclusions

In summary, this study suggests that poorer life satisfaction was associated with a dietary pattern characterized by foods high in free sugar and energy dense in adolescents, especially in girls aged 13 years of age. Since diet is a modifiable risk factor, the findings of this study may provide important implications for policy makers as well as identification of effective interventions. Given the fact of no previous studies on the associations between life satisfaction and dietary patterns among Malaysian young people, more studies are essential to support our findings. 

## Figures and Tables

**Table 1:** Characteristics of study adolescents recruited from three southern states of Peninsular Malaysia.

Variables	Boys			Girls			*p*-Value *
	n	Mean	SD	n	Mean	SD	
Overall life satisfaction	275	67.6	15.4	589	70.5	12.8	<0.05
Life satisfaction domains:							
Family	291	15.3	4.3	624	15.7	3.9	0.182
School	293	14.7	4.2	617	15.9	3.8	<0.001
Self	292	14.1	4.1	616	14.0	4.0	0.556
Friends	293	15.2	4.4	620	16.8	3.7	<0.001
Living environment	294	7.9	2.3	628	8.2	2.1	0.107
DP z-score	173	−0.15	1.14	411	0.06	1.40	0.060
Physical activity (PAQ-C score)	243	2.8	0.7	550	2.4	0.6	<0.001
	*n*	%		*n*	%		
Maternal education (*n* = 818)							
No formal education/ Primary school	23	9.5		68	11.8		0.584
Secondary school	179	74.3		425	73.7		
Higher institution	39	16.2		84	14.6		
School location (*n* = 933)							
Urban	147	49.0		287	45.3		0.295
Rural	153	51.0		348	54.7		

Scores for overall life satisfaction and for each domain were assessed using MSLSS; * *p*-value was estimated using independent t-test for continuous variables and chi-square for categorical variables.

**Table 2:** Adolescents’ responses on MSLSS life satisfaction items.

Domain	Item	*n*	Mean	SD	Disagree	Agree
					*n* (%)	
Family	1. My family is better than most.	922	3.80	1.41	150 (16.3)	772 (83.7)
	2. My parents treat me fairly.	926	4.06	1.27	125 (13.5)	801 (86.5)
	3. Members of my family talk nicely to one another.	927	4.02	1.26	116 (12.5)	811 (87.5)
	4. My parents and I do fun things together.	927	3.63	1.47	192 (20.7)	735 (79.3)
School	5. I look forward to going to school.	927	3.71	1.41	177 (17.0)	767 (83.0)
	6. School is interesting.	924	3.80	1.33	157 (17.0)	767 (83.0)
	7. I learn a lot at school.	922	4.05	1.16	99 (10.7)	823 (89.3)
	8. I enjoy school activities.	923	3.96	1.19	98 (10.6)	825 (89.4)
Self	9. There are lots of things I can do well.	924	3.95	1.14	93 (10.1)	831 (89.9)
	10. I think I am good looking.	922	3.05	1.55	293 (31.8)	629 (68.2)
	11. I like myself.	917	4.01	1.33	121 (13.2)	796 (86.8)
	12. Most people like me.	925	2.99	1.49	310 (33.5)	615 (66.5)
Friend	13. My friends are nice to me.	924	3.98	1.26	112 (12.1)	812 (87.9)
	14. My friends will help me if I need it.	924	3.93	1.24	107 (11.6)	817 (88.4)
	15. I have a lot of fun with my friends.	924	4.26	1.08	71 (7.7)	853 (92.3)
	16. I have enough friends.	922	4.09	1.24	100 (10.8)	822 (89.2)
Environment	17. I like my neighborhood.	922	3.70	1.40	168 (18.2)	754 (81.8)
	18. I like where I live.	926	4.37	1.12	68 (7.3)	858 (92.7)

Scores for each item in the domains were assessed using MSLSS.

**Table 3:** Relationships (β, 95% CI) between life satisfaction score and a “high sugar, high fibre, high DED and low fat” dietary pattern among adolescents aged 13 years in Malaysia.

Life Satisfaction	Boys (*n* = 169)				Girls (*n* = 379)			
	Unadjusted Beta Coefficient (95% CI)	Adjusted Beta Coefficient * (95% CI)			Unadjusted Beta Coefficient (95% CI)	Adjusted Beta Coefficient * (95% CI)		
		Model 1	Model 2	Model 3		Model 1	Model 2	Model 3
Overall score of life satisfaction	0.095 (−0.053, 0.216)	0.130 (−0.035, 0.256)	0.136 (−0.030, 0.267)	0.134 (−0.031, 0.266)	−0.090 (−0.100, 0.006)	−0.106 (−0.113, 0.000)	−0.108 (−0.118, 0.002)	**−0.119** **(−0.125, −0.004)**
Family	0.111 (−0.041,0.259)	0.147 (−0.025, 0.330)	0.144 (−0.040, 0.346)	0.143 (−0.041, 0.346)	−0.007 (−0.075, 0.066)	−0.003 (−0.076, 0.072)	−0.022 (−0.92, 0.062)	−0.024 (−0.094, 0.061)
School	**0.207** **(0.052, 0.322)**	**0.199** **(0.031, 0.341)**	**0.218** **(0.056, 0.366)**	**0.216** **(0.054, 0.364)**	−0.071 (−0.113, 0.018)	−0.073 (−0.188, 0.020)	−0.068 (−0.119, 0.028)	−0.079 (−0.126, 0.020)
Self	−0.030 (−0.175,0.118)	0.001 (−0.157, 0.158)	−0.020 (−0.078, 0.139)	−0.021 (−0.180, 0.139)	−0.093 (−0.136, 0.004)	**−0.122** **(−0.161, −0.013)**	**−0.128 ** **(−0.167, −0.014)**	**−0.131** **(−0.170, −0.015)**
Friends	0.105 (−0.047, 0.256)	0.057 (−0.117, 0.238)	0.070 (−0.111, 0.263)	0.069 (−0.113, 0.263)	−0.062 (−0.102, 0.023)	−0.076 (−0.116, 0.017)	−0.067 (−0.113, 0.028)	−0.068 (−0.114, 0.027)
Living environment	0.072 (−0.084, 0.236)	0.105 (−0.070, 0.293)	0.124 (−0.059, 0.331)	0.120 (−0.062, 0.326)	−0.097 (−0.140, 0.000)	**−0.108** **(−0.151, −0.004)**	**−0.114** **(−0.160, −0.004)**	**−0.119** **(−0.163, −0.007)**

* Model 1 was adjusted for school location and maternal educational level; * Model 2 was adjusted for school location, maternal education level, physical activity and BMI z-score; * Model 3 was adjusted for school location, maternal education level, physical activity, BMI z-score and dietary misreporting; Significant findings were bolded.
